# Mapping the diagnostic odyssey of congenital disorders of glycosylation (CDG): insights from the community

**DOI:** 10.1186/s13023-024-03389-2

**Published:** 2024-11-01

**Authors:** Pedro Granjo, Carlota Pascoal, Diana Gallego, Rita Francisco, Jaak Jaeken, Tristen Moors, Andrew C. Edmondson, Kristin A. Kantautas, Mercedes Serrano, Paula A. Videira, Vanessa dos Reis Ferreira

**Affiliations:** 1 UCIBIO – Applied Molecular Biosciences Unit, Department of Life Sciences, NOVA School of Science and Technology, Universidade NOVA de Lisboa, Caparica, Portugal; 2 Associate Laboratory i4HB - Institute for Health and Bioeconomy, NOVA School of Science and Technology, Universidade NOVA de Lisboa, Caparica, Portugal; 3 CDG & Allies-Professionals and Patient Associations International Network, Caparica, Portugal; 4 Portuguese Association for Congenital Disorders of Glycosylation (CDG), Lisbon, Portugal; 5 Centro de Diagnóstico de Enfermedades Moleculares, Centro de Biología Molecular-SO UAM-CSIC, Universidad Autónoma de Madrid, Campus de Cantoblanco, Madrid, Spain; 6 Centro de Investigación Biomédica en Red de Enfermedades Raras, Instituto de Investigación Sanitaria IdiPaZ, Madrid, Spain; 7 Center for Metabolic Diseases, Department of Pediatrics, KU Leuven, Leuven, 3000 Belgium; 8 Glycomine, Inc, 733 Industrial Road, San Carlos, CA 94070 USA; 9 Division of Human Genetics, Department of Pediatrics, Children’s Hospital of Philadelphia, Philadelphia, PA USA; 10 Perlara PBC, Berkeley, CA 94705 USA; 11 Neurology Department, Hospital Sant Joan de Déu, U-703 Centre for Biomedical Research on Rare Diseases (CIBER-ER), Instituto de Salud Carlos III, Barcelona, Spain

**Keywords:** Congenital disorders of glycosylation (CDG), Patient journey, Diagnostic odyssey journey, Community-centered research, Rare diseases

## Abstract

**Background:**

Congenital disorders of glycosylation (CDG) are a group of rare metabolic diseases with heterogeneous presentations, leading to substantial diagnostic challenges, which are poorly understood. Therefore, this study aims to elucidate this diagnostic journey by examining families’ and professionals’ experiences.

**Results and discussion:**

A questionnaire was designed for CDG families and professionals, garnering 160 and 35 responses, respectively. Analysis revealed the lack of seizures as a distinctive feature between PMM2-CDG (11.2%) with Other CDG (57.7%) at symptom onset. Hypotonia and developmental disability were prevalent symptoms across all studied CDG. Feeding problems were identified as an early onset symptom in PMM2-CDG (Cramer’s V (V) = 0.30, False Discovery Rate (FDR) = 3.8 × 10^− 9^), and hypotonia in all studied CDG (V = 0.34, FDR = 7.0 × 10^− 3^). The average time to diagnosis has decreased in recent years (now ~ 3.9 years), due to advancements namely the increased use of whole genome and exome sequencing. However, misdiagnoses remain prevalent (PMM2-CDG – 44.9%, non-PMM2-CDG – 64.8%). To address these challenges, we propose adapting medical training to increase awareness of CDG and other rare diseases, ongoing education for physicians, the development of educational resources for relevant medical units, and empowerment of families through patient organizations and support networks.

**Conclusion:**

This study emphasizes the crucial role of community-centered research, and the insights families can offer to enhance CDG management. By pinpointing existing gaps and needs, our findings can inform targeted interventions and support systems to improve the lives of those impacted by CDG.

**Supplementary Information:**

The online version contains supplementary material available at 10.1186/s13023-024-03389-2.

## Background

The “Patient Journey” is the sequence of events that a patient undergoes while receiving healthcare services [1]. This journey can be divided into five stages: (1) onset of symptoms; (2) diagnosis and building of a therapeutic relationship with healthcare professionals; (3) initiation of care; (4) clinical management; and (5) living with the disease (if chronic) [2]. Of particular importance within these stages is the diagnostic journey, which involves the patient’s initial encounter with a healthcare provider, the first round of referrals, and subsequent evaluations by additional specialists to arrive at an accurate diagnosis [3].

Rare diseases, affecting < 1 person in 2000 according to the European Union definition [4], often have a long and complicated diagnostic journey, which can take an average 5 years [5]. The primary care referral often requires extensive testing and multiple visits to different specialists, sometimes leaving patients in this referral loop for years [3, 6, 7]. Moreover, the lack of awareness, coordination, and management among healthcare professionals can make these diagnostic loops even more complex, leading to a series of negative outcomes. These can include both direct and indirect costs, significant amount of stress for families, misdiagnoses, avoidable medical errors and instances of iatrogenesis - that is to say, deleterious effects attributable to medical intervention [8]. All these factors can lead to obstacles in patients’ access to appropriate treatment options. This issue is of utmost importance, as starting the most appropriate treatment earlier is usually linked with better health outcomes [9]. Furthermore, the post-diagnosis period is often marked by uncertainty, with many questions regarding the future. This often leads to demands from families for more detailed information from healthcare providers or self-education if they feel a lack of adequate guidance [10].

In this study, we focus on Congenital Disorders of Glycosylation (CDG), a heterogeneous group of around 170 rare genetic metabolic diseases with an estimated overall prevalence of 0.1–0.5 per 100,000 in Europe [11, 12]. They are caused by defects in the genes involved in the biosynthesis of glycoconjugates [13]. These defects ultimately affect the biological functions of glycoconjugates spanning structural (e.g., protein stability and folding), energy metabolism (e.g., nutrient availability) and informational (e.g., receptor recognition and signaling) roles [14]. CDG can be categorized into four primary groups, according to their inherent abnormalities: (a) N-linked glycosylation, (b) O-linked glycosylation, (c) combined N- and O- linked or multiple glycosylation pathways (most predominant number of phenotypes), and (d) lipid and glycosylphosphatidylinositol (GPI) anchor defects [15]. CDG often have severe, multi-organ symptomatology, usually involving the central nervous system, and few treatment options are available [16–22]. PMM2-CDG is the most common CDG, with more than 1000 patients recorded to date. Phenotypes range from mild to severe, including neonatal death [23]. The number of CDG and their associated phenotypes is increasing, and the lack of specific biomarkers for initial suspicion for every CDG, the unawareness or even lack of technical capabilities to analyze recently identified biomarkers, makes diagnosis challenging. For example, the standard transferrin isoelectric focusing (TIEF) test, a traditional diagnostic method, is limited in scope, detecting most N-linked glycosylation anomalies that lead to deficiency of sialic acid [24]. Beyond this “gold standard” test, there is a growing list of other CDG biomarkers, such as O-xylose glycosaminoglycan defects in bikunin for B3GAT3-CDG, B4GALT7-CDG, CCDC115-CGD, among other CDG, and defects in mucin core1 O-glycosylation in Apolipoprotein C-III for Golgi-apparatus impairment (e.g., COG-CDG, ATP6VOA2-CDG, CCDC115-CDG and GALNT2-CDG) [25, 26].This lack of initial clinical suspicion makes molecular testing for various CDG essential for diagnosis, though such tests may not be easily available in all regions. Consequently, numerous patients remain undiagnosed and untreated [12, 27]. Therefore, understanding the CDG journey is vital, as it helps identify existing gaps in healthcare and addresses the lack of awareness and knowledge about these diseases, particularly within the medical community. In-depth insights into the journey and challenges of CDG not only enhances patients’ experiences but also may help reduce the economic and emotional strain associated with diagnostic delays. It can also respond to families’ informational needs and promotes a more patient-centered approach to care [2, 28].

The present research is designed to illustrate the experiences of those living with CDG, along with their caregivers and professionals, specifically focusing on the diagnostic journey. As noted in prior work, the CDG community actively participates in collaborative initiatives [29, 30]. Hence, we co-developed an electronic survey to collect information about the patient’s path through the healthcare system, starting with the search for a diagnosis and continuing thereafter. Our multi-stakeholder approach aims to shed light on the current healthcare system’s operation and identify its shortcomings in diagnosing CDG. Our ultimate goal is to enhance the healthcare experience and quality of life for CDG patients and their families.

## Methods

### Co-development and refinement of the CDG journey mapping questionnaire

The CDG Journey Mapping Electronic Questionnaire had two adapted versions designed to include two target audiences from various backgrounds. One version was intended for individuals with CDG, their family members, and/or caregivers. Another version was geared toward professionals (healthcare providers and researchers).

Both versions of the survey had undergone development and refinement, using a literature review and input from members of the CDG community. Additionally, an advisory committee of 16 individuals, consisting of professionals and family members, provided valuable input during the development process, as previously described [30].

The questionnaire was composed of 96 and 79 questions in the family and professional versions, respectively. Aside from minor exceptions, both versions of the survey focused on similar themes from different perspectives. The professional version was only available in English, whereas the family version was available in English, Spanish, Portuguese, and Italian.

For this study, questions were selected from various themes with the specific aim of uncovering the CDG diagnostic journey. The questions exhibit minor differences between those aimed at families (Additional File 1) and those aimed at professionals (Additional File 2).

The study received ethical approval from the ethics committee of the Faculty of Psychology at the University of Lisbon, and all participants provided informed consent electronically. The e-format of the questionnaire was applied using the SurveyMonkey Audience platform (www.surveymonkey.com/mp/audience — Copyright © 1999–2023 Momentive). To ensure the respondents’ anonymity, their IP addresses were not recorded. The restriction on multiple entries was enabled. Multiple-choice, matrix, and open-ended question formats were used. To lessen the burden on the participants, a logic feature was added to specific questions, which also helped guide the participants.

### Questionnaire dissemination

The online e-questionnaire was open for 5 months from May to October of 2021. The surveys were distributed as previously described [29–32]. Briefly, several strategies were used to reach the intended target audience, namely direct messaging via email retrieved from the World CDG Organization database as well as various social media channels. These platforms included WhatsApp, Facebook, and Twitter from a variety of sources, including Patient Organizations (CDG CARE, Sindrome Brasil, and the Portuguese Association for CDG (APCDG), among others) and international networks (Frontiers in CDG Consortium (FCDGC) and MetabERN). Also, a webpage dedicated to this project was created on the worldcdg.org website acting as a central dissemination channel.

### Inclusion criteria

The inclusion criteria for this study were strictly limited to participants who were 18 years old or older. Additionally, we only considered family members and caregivers who have a direct relationship with a person who has a confirmed CDG diagnosis. We rigorously screened the responses to ensure the integrity of our data. We excluded responses under several conditions: suspected duplicates (*n* = 7), absence of a confirmed CDG diagnosis (*n* = 5), involvement with NGLY1 defects (*n* = 3), and inconsistencies in responses (*n* = 4). We were unable to identify patients who had already died, and the potential testimonies from their families on this questionnaire. This limitation, coupled with a lack of clarity regarding this topic, might have resulted in the loss of this small cohort. Moreover, patients with GNE Myopathy (GNE-CDG) (*n* = 14) were deliberately excluded from this study. The rationale for this exclusion was based on the distinct nature of their disease journey, adult onset and organ specific disease, which we determined necessitated a separate analytical focus. This decision was made to ensure a more homogeneous study population and to maintain the specificity of the insights derived from our research. From all this, out of the 193 responses received from the families’ version, 160 were selected for inclusion in this study. Concerning clinical-related questions, we only considered responses provided by professionals with experience in diagnosing or assessing patient outcomes (healthcare professionals and clinicians). This included 35 responses from the professionals’ version.

### Statistical analysis

For this study, we performed statistical analyses using R software (version 4.1.1) and Microsoft Excel 2021, while GraphPad Prism version 8 was used to create graphs. As most of the variables in this study were categorical, we use descriptive statistics to analyze and report our findings. To determine associations between categorical variables, we used Fisher’s exact test, with all p-values corrected for multiplicity using the False Discovery Rate (FDR) method, if necessary. We conducted post-hoc tests, including odds ratio (OR), phi coefficient, cramer’s V (V), if FDR ≤ 0.05. The psych (version 2.2.9), confintr (version 1.0.2) and FactoMineR (version 1.0.7) packages were utilized for these tests [33–35]. Additionally, we conducted a Multiple Correspondence Analysis (MCA) using the FactoMineR package to evaluate the involvement of multiple frequent signs and symptoms at the onset.

## Results

### Participant demographics

**Fig. 1:**
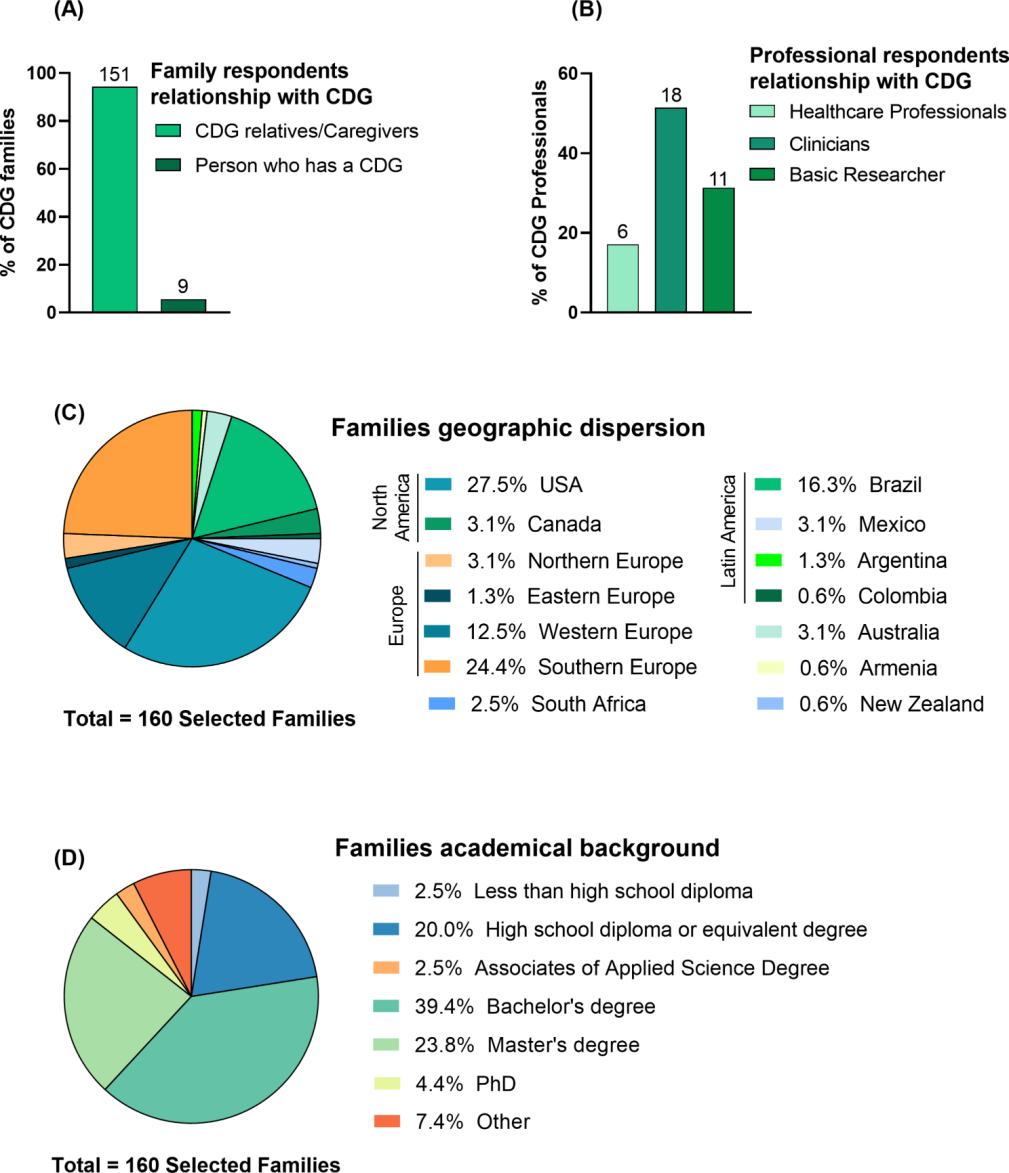
Socio-demographic Profile of Study Participants: (**A**) Families and (**B**) Professionals’ Relationship to the CDG Community, (**C**) Families geographic dispersion, and (**D**) Academic qualifications

In this study, we included a total of 160 CDG relatives/family caregivers and people living with CDG, from now on referred as “CDG families”, and 35 CDG professionals, including healthcare professionals, clinicians, and basic researchers. The CDG families’ group was composed of 151 CDG relatives/caregivers (94.4%) and 9 people who has CDG participating (5.6%, Fig. 1A). In the CDG professionals’ group, clinicians were the most represented (51.4%, *n* = 18) followed by basic researchers (31.4%, *n* = 11) and healthcare professionals (17.2%, *n* = 6) (Fig. 1B).

The study had a diverse distribution of participants among CDG families and professionals, with a higher incidence of individuals between the ages of 35–54 in both groups (68.8%, *n* = 110 and 57.1%, *n* = 20, respectively), and from the female gender (88.1%, *n* = 141 and 62.9%, *n* = 22, respectively; Additional File 3). In terms of geographical dispersion, the largest proportion of family respondents were from Europe (41.3%, *n* = 66), followed by North America (30.6%) and Central and Southern America (Latin America), making up 21.3% (*n* = 34). Smaller numbers of respondents were from other countries like Australia (3.1%; *n* = 5), South Africa (2.5%; *n* = 4), Armenia (0.63%; *n* = 1) and New Zealand (0.63%; *n* = 1, Fig. 1C). Further details on the distribution of respondents are described on Additional File 3. Professionals display a similar geographic distribution as described by Francisco et al. [30] Concerning their academic qualifications, 67.6% (*n* = 108) participants from the CDG families’ group had at least a bachelor’s degree (Fig. 1D), while 68.6% (*n* = 22) of professionals hold a PhD, indicating a high level of education in both groups.

### Clinical characterization of CDG participants

**Fig. 2:**
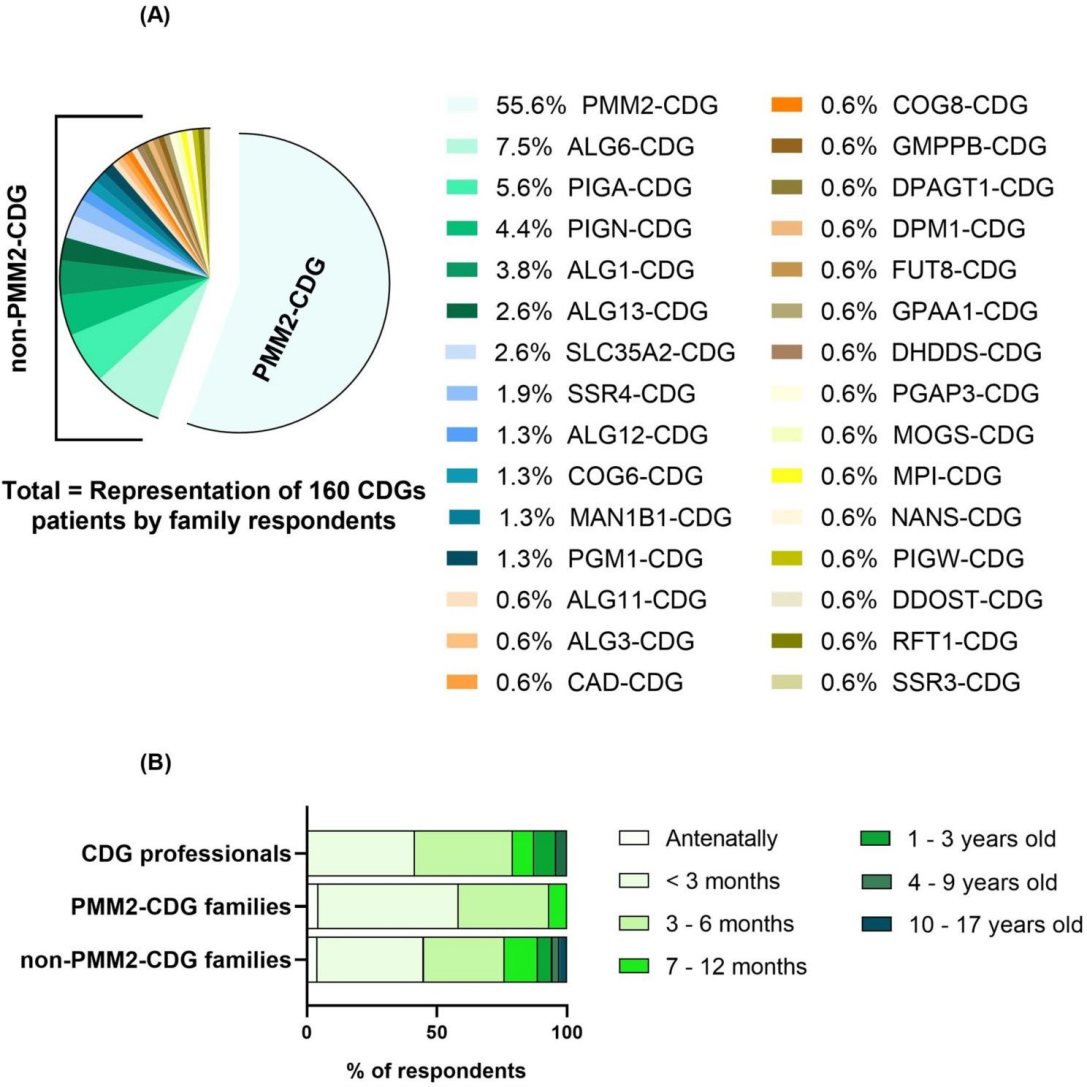
CDG clinical characterization: (**A**) Distribution of CDG as reported by participating families, (**B**) Perceived age of symptom onset in CDG patients

The CDG families’ group represented a total of 30 genetic causes of CDG, including 16 N-linked glycosylation defects, 5 GPI biosynthesis defects, and 9 disorders of multiple glycosylation pathways. O-linked glycosylation defects and lipid glycosylation defects were not reported (Additional File 3). PMM2-CDG (MIM: 212065) was the most common CDG (55.6%, *n* = 89), followed by ALG6-CDG (MIM: 603147; 7.5%, *n* = 12) and PIGA-CDG (MIM: 300868; 5.6%, *n* = 9; Fig. 2A). The frequency of each CDG among respondents, reflects the worldwide prevalence these diseases [12, 36, 37]. Due to the overrepresentation of PMM2-CDG and in order to gain a thorough understanding of the differences between this and other CDG regarding the clinical-related aspects of their journey, the CDG families group was divided into two, namely PMM2-CDG and non-PMM2-CDG, in line with previous studies [29, 38].

Signs and symptoms onset is the first hallmark of a patient’s journey, and the nature of their manifestations can vary depending on the CDG. Our findings showed that all three groups included in our study reported that CDG typically manifested before the first year of life, most often even before the age of 6 months old (PMM2-CDG − 93.3%, *n* = 83, non-PMM2-CDG − 76.1%, *n* = 54, Professionals − 79.2%, *n* = 19, Fig. 2B).

We identified a range of clinical signs and symptoms at onset in each CDG (Additional File 4). Several symptoms were observed to have a frequency of at least 50% in PMM2-CDG or non-PMM2-CDG groups, aligning with responses from the professionals’ group. However, other symptoms were recorded with lower frequencies, falling below the 50% threshold (Table 1). Hypotonia and developmental disability were consistently reported as the most common onset symptoms across all groups. Notably, people with PMM2-CDG demonstrated a slightly higher percentage of hypotonia and developmental disability compared to non-PMM2-CDG patients. In contrast, seizures were also identified as a primary symptom by both professionals and the non-PMM2-CDG group, but not by those with PMM2-CDG, as evidenced by the lower OR when compared to the non-PMM2-CDG. Furthermore, feeding problems, ataxia, and strabismus were frequently observed at the onset for the PMM2-CDG group, with a significantly higher OR for these symptoms in this group compared to the non-PMM2-CDG group. Concerning the frequency of these symptoms, some inconsistencies were noted in the professionals’ group when compared with the other two groups. The percentage of feeding problems was more similar to the PMM2-CDG group, while strabismus and ataxia were more aligned with the non-PMM2-CDG group.

In order to understand the incidence of a combination of symptoms in people with CDG during symptom onset, a MCA was performed. This MCA aimed to verify the potential unreported relationships of clinical presentations during the onset. Also, it is important to characterize a general profile of a person living with CDG, apart from the heterogeneity that is seen within CDG. Therefore, besides the PMM2-CDG and non-PMM2-CDG groups, we also considered the whole CDG family’s group to identify potential common traits and signs and symptoms (Additional File 5: Fig. 1). All three groups display a combination of symptoms, but seizures did not aggregate with the other onset symptoms in PMM2-CDG compared to non-PMM2-CDG. Thus, seizures are less frequently part of the onset of PMM2-CDG patients which is also clear from the overall prevalence of this symptom in this CDG.

We also aimed to examine any associations between the time of manifestation and specific symptoms, with a particular focus on the identification of early onset symptoms in CDG. Early onset in this clinical context typically refers to symptom manifestation in the first months of life (< 3 months). To do this, we used the time of manifestation and the main identified onset symptoms as reference points. We found some associations between certain symptoms and their onset in PMM2-CDG and the whole CDG families group. In PMM2-CDG, there was a weak association between feeding problems and earlier onset (V = 0.30, FDR = 3.8 × 10^− 9^). For the CDG family’s group, we observed a weak association between an earlier onset and hypotonia (V = 0.34, FDR = 7.0 × 10^− 3^).

**Table 1:** Number of patients presenting with specific symptoms at onset in two subcohorts: PMM2-CDG and non-PMM2-CDG, along with professional perspectives

Feature	PMM2-CDG		non-PMM2-CDG		FDR *	OR	Phi	Professionals	
Number of patients	89		71		-			24	
	n	%	n	%				n	%
**Hypotonia**	78	**87.6**	54	**76.1**	0.13	-	-	22	**91.7**
**Developmental disabilities**	73	**82.0**	48	**67.6**	0.098			22	**91.7**
**Strabismus**	61	**68.5**	20	28.2	2.9e^− 6^	5.47	0.4	8	33.3
**Ataxia**	49	**55.1**	16	22.5	1.1e^− 4^	4.15	0.33	4	16.7
**Feeding Problems**	46	**51.7**	13	18.3	8.9e^− 5^	4.69	0.33	11	45.8
Seizures	10	11.2	41	**57.7**	4.6e^− 9^	0.095	0.5	14	**58.3**
Poor growth	37	41.6	14	19.7	1	-	-	12	**50**
Failure to thrive	33	37.1	14	19.7	1			19	**79.2**
Liver Disease/and or elevated liver enzymes	18	20.2	5	7	1			10	41.7
Blood Abnormalities	2	2.2	2	2.8	1			4	16.7
Stroke-like episodes	6	6.7	4	5.6	1			1	4.2
Heart Problems	6	6.7	2	2.8	1			2	8.3
Dysarthria	14	15.7	4	5.6	1			0	0
Bone manifestations	8	9	4	5.6	1			1	4.2
Abnormal brain imaging	24	27	9	12.7	1			5	20.8
Abnormal lab tests	18	20.2	6	8.5	1			8	33.3
Recurrent infections	11	12.4	11	15.5	0.94			0	0
Poor night vision and loss of peripheral vision	8	9	1	1.4	1			11	45.8

Symptoms reported by more than 50% of the respondents from each group are highlighted in bold

* denotes the false discovery rate (FDR) obtained from the multiplicity adjustment of every Fisher Test from this table. The Odds Ratio (OR) and Phi Coefficient (Phi) depict the strength of each association

### The encounter with healthcare professionals and the time to arrive at a diagnosis

**Fig. 3:**
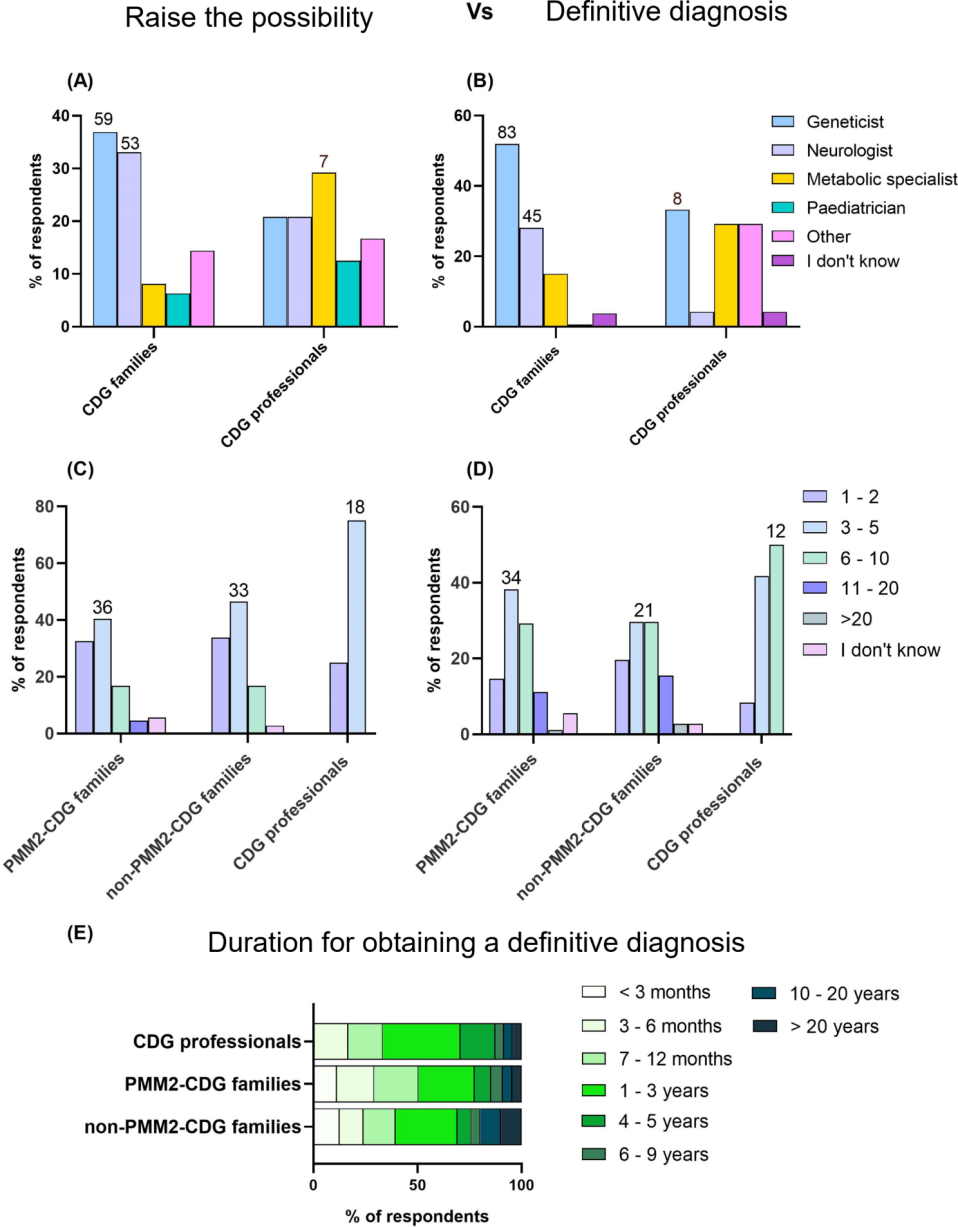
Healthcare Professionals Involved in CDG Diagnostic Journey: (**A**) Specialists Raising CDG Possibility, and (**B**) Giving Final Diagnosis, both from the insights of families and professionals. Number of Doctors that families faced to raise the possibility of a CDG and between this first possibility and the definitive diagnosis, (**C**) Symptoms Onset to Raise the Possibility, (**D**) Time from Suspicion to Definitive Diagnosis, and (**E**) Estimated Time to Definitive Diagnosis

CDG families were asked who raised the possibility of a CDG diagnosis and who provided the final diagnosis (Fig. 3A, B). In both cases, the most common experts involved who contributed to the final diagnosis were geneticists (raise the possibility − 36.9%, *n* = 59; definitive diagnosis − 51.9%, *n* = 83) and neurologists (raise the possibility − 33.1%, *n* = 53; definitive diagnosis − 28.1%, *n* = 45). Professionals, on the other hand, reported a broader distribution of potential specialists for both situation, with metabolic specialists being the most likely to raise the suspicion of a CDG diagnosis (29.2%, *n* = 7) and geneticists the definitive diagnosis (33.3%, *n* = 8).

To get a full picture of the participants’ quest for a diagnosis, we inquired about the number of doctors consulted. Firstly, our data shows that it usually took at least the consultation of 3 to 5 doctors between the onset of signs and symptoms and raising the possibility of a CDG diagnosis (PMM2-CDG – 73.0%, *n* = 65; non-PMM2-CDG – 80.3%, *n* = 57; Professionals − 75.0%, *n* = 18) (Fig. 3C, D). Secondly, between this first suspicion to a definitive diagnosis, it was common to consult at least 3 to 5 doctors (PMM2-CDG – 52.8%, *n* = 47; non-PMM2-CDG – 49.3%, *n* = 35; professionals − 41.7%, *n* = 12) or more. In fact, 6 to 10 doctors were required to get a final diagnosis in 29.4% (*n* = 26) of the PMM2-CDG and in 29.6% (*n* = 21) of non-PMM2-CDG patients. The percentage of respondents that stated this interval is even higher from the professionals’ perspective (41.7%, *n* = 10).

Regarding the time to get a definitive CDG diagnosis, approximately a third of the professionals (37.3%, *n* = 9) stated that patients usually have their diagnosis between the age of 1–3 years. Likewise, approximately a third of PMM2-CDG (27.0%, *n* = 24) and non-PMM2-CDG (29.6%, *n* = 21) families had indicated the same. Notably, 50.5% (*n* = 45) of the PMM2-CDG and 39.5% (*n* = 28) of the non-PMM2-CDG groups received their diagnosis in less than a year (Fig. 3E). However, there were still patients for whom the diagnosis took more than 4 years (Additional File 3).

Also, the participants’ country of living might influence the number of medical doctors consulted before the diagnosis was made. To address this, our cohort was divided into four groups: North America (30.6%, *n* = 49), Europe (41.3%, *n* = 66), Latin America (21.3%, *n* = 34) and Other-countries (6.8%; *n* = 11) (Additional File 5: Fig. 2). Although no statistical differences were found, there is a noticeable trend in the number of doctors consulted between the first suspicion to the definitive diagnosis of CDG across these groups. In Latin America, there is a tendency for patients to consult more doctors (6–10 doctors) with 47.1% (*n* = 75) falling into this group. In contrast, in Europe, 45.5% (*n* = 30) of individuals sought fewer doctors (3–5 doctors) during the same period. (Additional File 5: Fig. 2B). Regarding the time to obtain a definitive diagnosis, no statistical differences were found, but slight variations can be seen between groups. In Europe, 53.0% (*n* = 35) of respondents were able to get a definitive diagnosis in less than a year, compared to 40.8% (*n* = 26) in North America, 29.4% (*n* = 10) in Latin America, and 73.6% (*n* = 8) in Other Countries. In Latin America, most respondents (47.1%; *n* = 16) received their diagnosis within 1–3 years. Furthermore, it important to point out that across all groups, there were cases where diagnosis took more than 3 years, up to 20 years.

Another key factor that could potentially accelerate the diagnostic journey is the possibility of testing for CDG by the serum TIEF test, and its results normal or abnormal (type 1 or type 2 pattern, Additional File 3) [27]. By a similar analysis, we found no significant differences between the normal TIEF (*n* = 24) and abnormal TIEF (*n* = 136) groups (Additional File 5: Fig. 3).

### Perception of CDG families regarding diagnosis accuracy

**Fig. 4:**
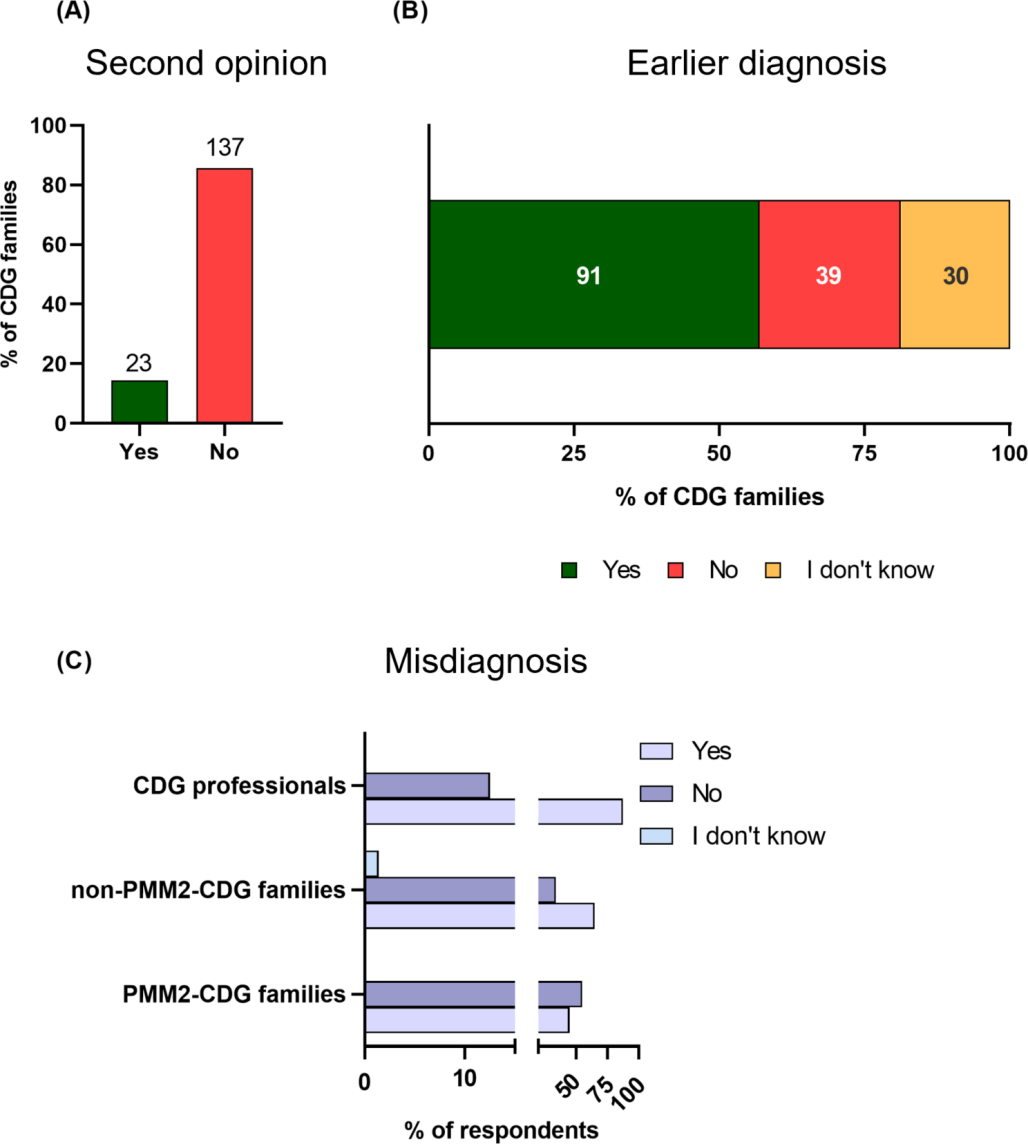
Illustration of a patient’s journey through the healthcare system. Perceptions of families (PMM2-CDG and non-PMM2-CDG) and professionals: (**A**) Misdiagnosis (Differences in the presence or absence of Misdiagnosis between groups with a FDR = 0.014, Odds Ratio: 2.3 and Phi Coefficient: 0.21, (**B**) Families who sought a second opinion following a CDG diagnosis, (**C**) Families’ opinions on the overall time taken to obtain their diagnosis

The majority of the CDG families demonstrated a general acceptance of the final CDG diagnosis, as 85.6% of respondents did not seek a second opinion (Fig. 4A). This finding indicates a high level of trust in the initial diagnostic process among the participating families. Nevertheless, a considerable proportion of families (56.9%, *n* = 91) expressed the belief that the diagnosis could have been reached at an earlier stage. In contrast, 18.8% (*n* = 30) of the families reported uncertainty regarding the potential for an earlier diagnosis, while 24.4% (*n* = 39) had the opinion that an earlier diagnosis was not feasible (Fig. 4B).

In rare diseases, there is an increase in misdiagnoses largely due to the lack of awareness and knowledge among healthcare professionals [39–41]. Concerning misdiagnoses, 87.5% (*n* = 21) of professionals stated that CDG patients normally have misdiagnoses, and 64.8% (*n* = 46) and 44.9% (*n* = 40) of the non-PMM2-CDG and PMM2-CDG groups, respectively, reported a misdiagnosis during their diagnosis-seeking journey (Phi = 0.21, OR = 2.3, FDR = 0.014, Fig. 4C). However, the type of misdiagnosis may differ significantly between and within each CDG (Additional File 3). There were also some differences between countries where Europe seems to have a lower number of misdiagnoses in comparison to the other countries worldwide (FDR = 0.025, V = 0.24, Additional File 5: Fig. 2D).

### Journey of family with multiple CDG diagnosis

**Fig. 5:**
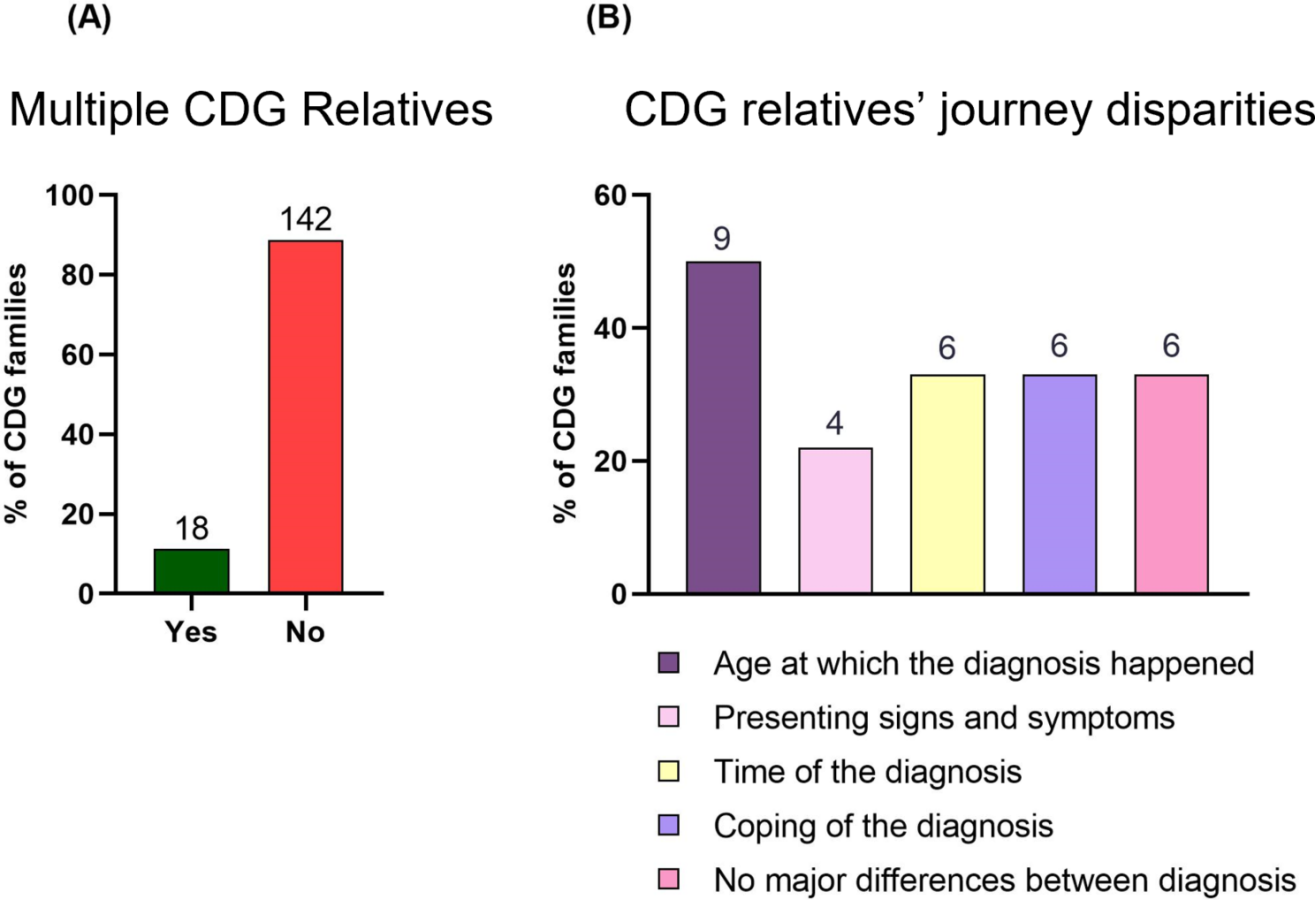
Families with multiple CDG relatives (**A**) Overall prevalence of Multiple CDG relatives in the stakeholder group of families, (**B**) Identified differences between family members (Multi-Choice Close Question)

As CDG are genetic and mostly inherited diseases, it was important to determine whether there were families in our cohort who had multiple offspring with CDG, and to learn about their experiences (Fig. 5). In the present study, 11.3% (*n* = 18) of the families had more than one relative with CDG (Fig. 5A), encompassing 9 families with PMM2-CDG, 2 with ALG6-CDG (*n* = 2/18), and 1 with FUT8-, ALG1-, PIGN-, DDOST-, PGM1-, PIGA-, and ALG11-CDG. The most pronounced difference between the CDG relatives’ journey was the age of diagnosis (*n* = 9/18) (Fig. 5B). One third (*n* = 6/18) of the respondents stated that there were no significant differences, while another third (*n* = 6/18) stated that there was a difference in coping (e.g., information needs, and emotional impact), and/or time of diagnosis. Also, 4 families reported differences in the presenting signs and symptoms.

### Information sources and formats

**Fig. 6:**
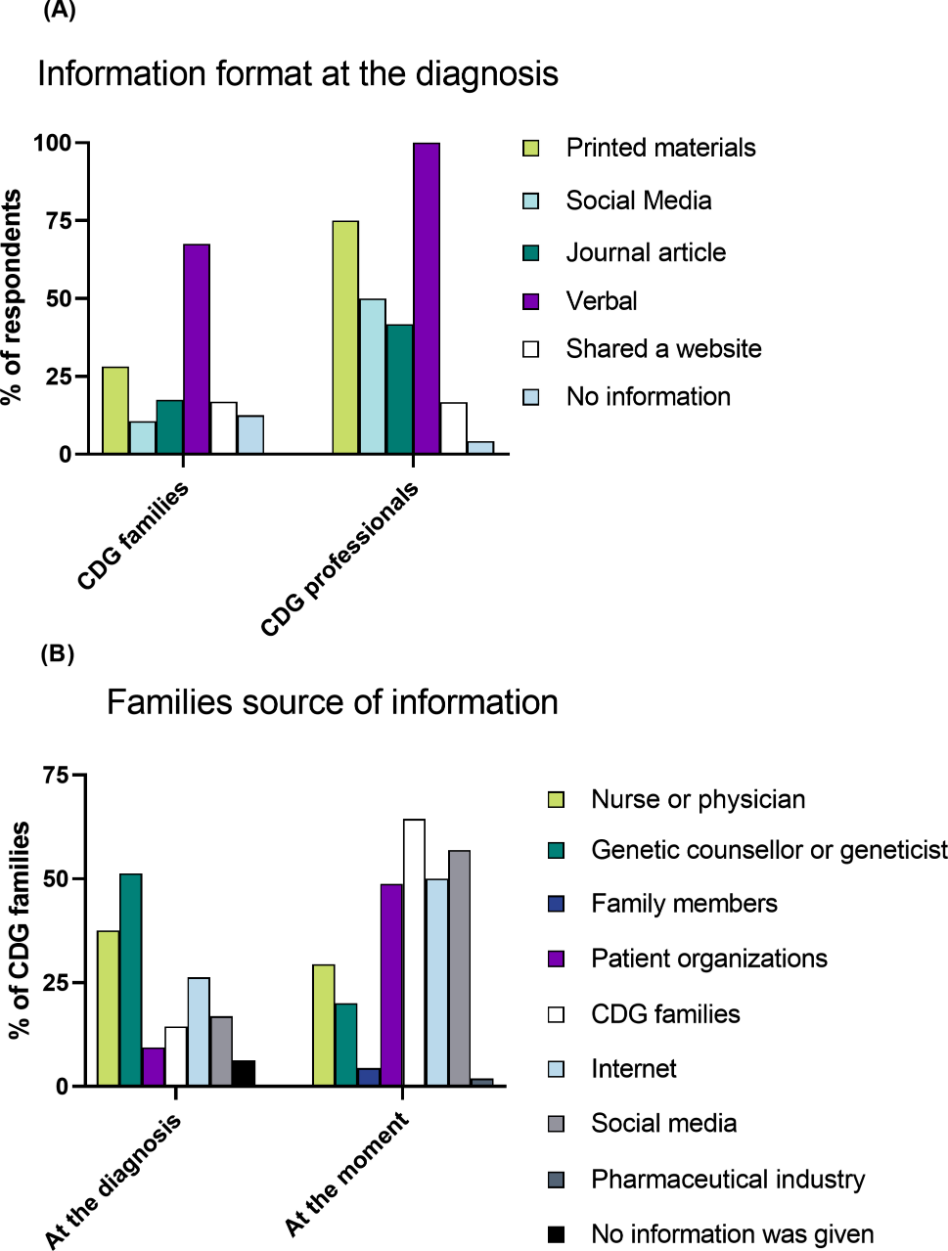
Assessment of Patients’ Access to CDG Information during Diagnostic Journey: (**A**) Information Format at Time of Diagnosis, and (**B**) Information Sources for Families at Diagnosis and Present

Given the rarity of CDG and the frequent lack of available information, this study sought to examine the informational needs of CDG families from both the perspectives of the families themselves and the professionals involved in their care. We specifically asked about the format in which CDG-related information was provided at the time of diagnosis, as well as what was and currently is their primary source of information (Fig. 6).

At the time of diagnosis (Fig. 6A), verbal communication was identified as the most common format for conveying information by both professionals (100%, *n* = 24) and families (67.5%, *n* = 108). Professionals with experience in CDG diagnosis indicated that they share leaflets (75%, *n* = 18) and social media channels (50%, *n* = 12) as supplementary sources of information for CDG families. However, only a small proportion of families received information through any of these two formats (leaflets − 28.1%, *n* = 45; social media – 10.6%, *n* = 17).

During the diagnostic period, families primarily sought information from genetic counselors and geneticists (51.3%, *n* = 82) and healthcare professionals (37.5%, *n* = 60, Fig. 6B). Over time, there has been a shift towards other sources, such as fellow CDG family members (64.4%, *n* = 103), social media (56.9%, *n* = 91), and Patient Organizations (POs) (48.8%, *n* = 78).

**Fig. 7:**
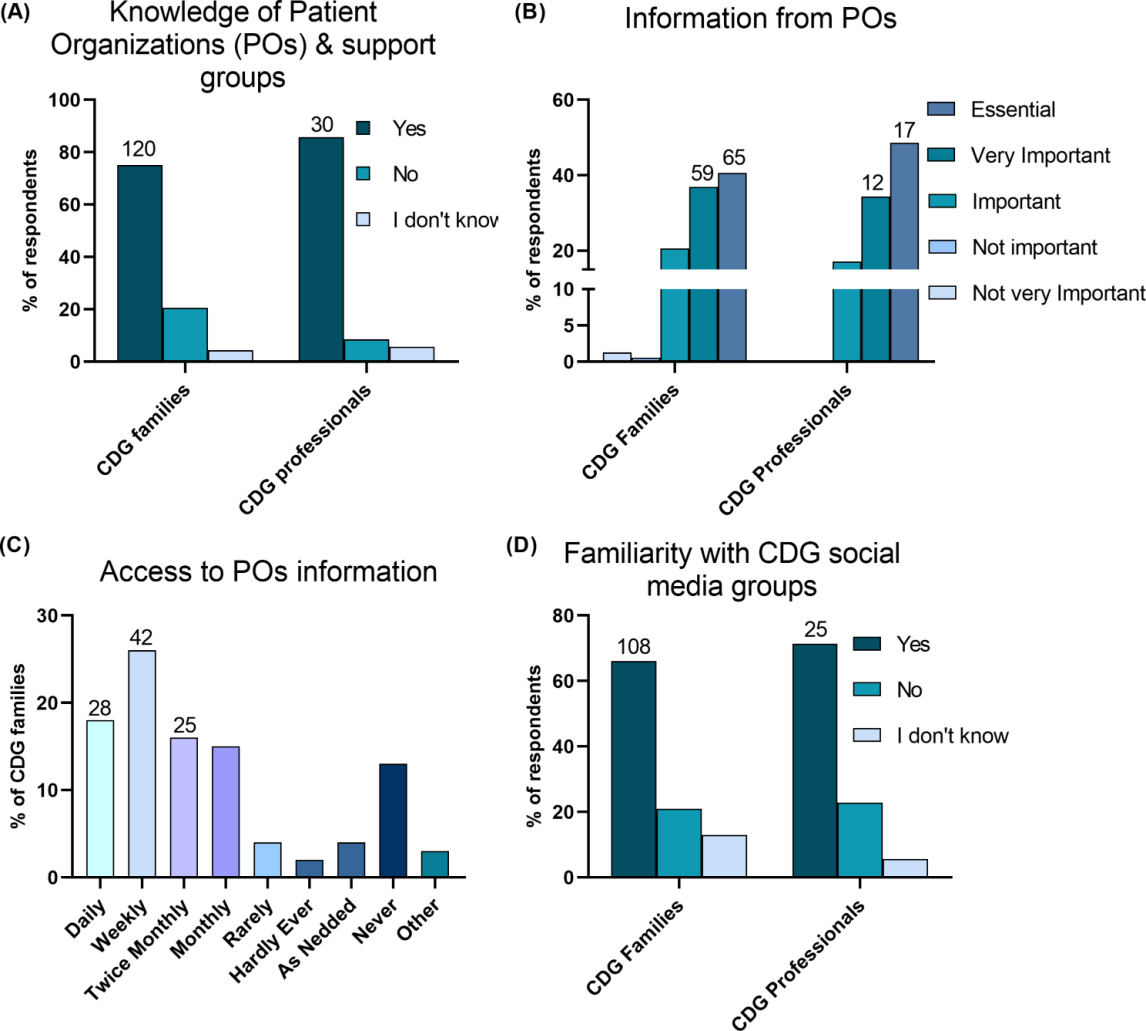
Depiction of the importance of CDG-specific support groups for families. (**A**) Awareness of the existence of CDG-specific Patient Organizations (POs) among families and professionals, (**B**) Recognition of the value of information provided by POs. from both family and professional perspectives. (**C**) Family access to POs-tailored information. (**D**) Familiarity with CDG social media groups among families and professionals

POs and support groups are extremely important for rare disease communities like CDG. Therefore, we assessed respondents’ knowledge of CDG-related POs and support groups (Fig. 7A). The majority of both groups (Families − 75.0%, *n* = 120; Professionals − 85.7%, *n* = 30) reported being aware of at least one CDG-oriented organization or support group.

Considering the established role of support groups and POs as information providers [42], we aimed to elucidate the significance of these organizations for the families and professionals in our cohort. In regard to POs, both groups deemed the provided information to be either Very important (Families − 36.9%, *n* = 59; Professionals − 34.3%, *n* = 12) or Essential (Families – 40.6%, *n* = 65; Professionals − 48.6%, *n* = 17) for the community (Fig. 7B). Additionally, nearly half of the families (43.8%, *n* = 70), access this information on a weekly basis, with only a small percentage accessing it Rarely (4%, *n* = 6) or Never (13%, *n* = 21) (Fig. 7C).

Social media has emerged as a versatile tool with various applications, including information dissemination and support group formation. Our study surveyed participants to determine their familiarity with CDG social media groups, revealing that a considerable proportion of respondents were acquainted with such groups (67.5% of families, *n* = 108; and 71.4% of professionals, *n* = 25) (Fig. 7D). Furthermore, both groups identified potential opportunities associated with social media. While a substantial number of prospective opportunities were documented (Additional File 5: Fig. 4), there was a consensus that increasing CDG awareness holds the most potential in the context of social media platforms.

## Discussion

**Fig. 8:**
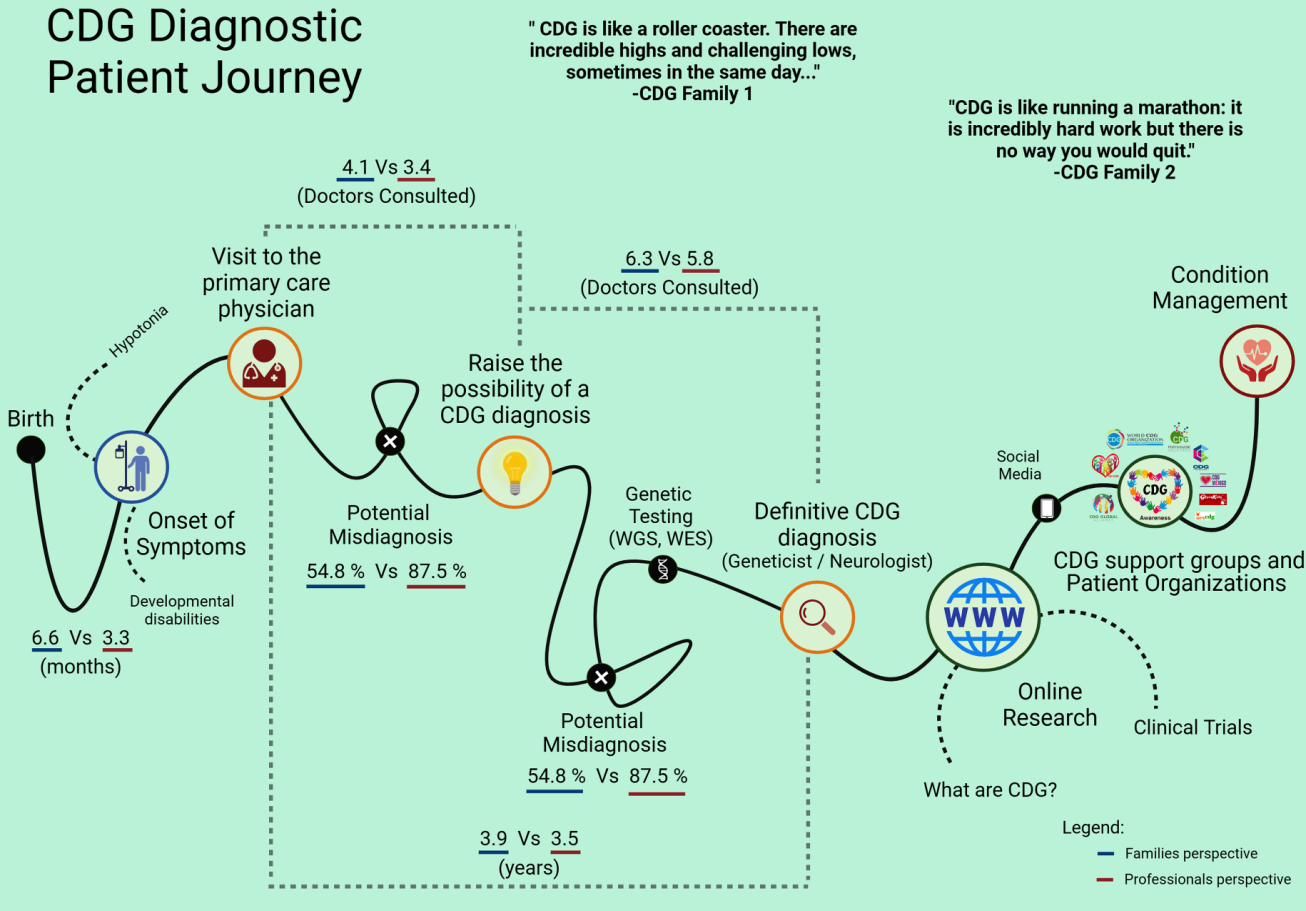
Depiction of the CDG diagnostic journey based on the results made by families and professionals

When CDG are diagnosed, families often face a “diagnostic odyssey” due to a lack of information and support [43–45]. CDG are especially challenging because they comprise many different diseases, most with their own characteristics [27]. To address this issue, we developed the CDG Journey Mapping questionnaire, designed to gather both the perspectives of families and professionals, resulting in an organized and consistent overview of the CDG diagnostic journey (Fig. 8).

The CDG diagnostic journey begins with the onset of symptoms. Developmental disability and hypotonia are the most common neurological symptoms across all CDG included in our study, which aligns with prior research, among other notable differences between PMM2-CDG and non-PMM2-CDG groups [46–52]. Our findings suggest that seizures may be a distinctive feature between PMM2-CDG and other CDG, potentially aiding in the diagnostic phase. This is due to the lower prevalence of seizures in the PMM2-CDG group, and the significantly reduced likelihood of seizures manifesting at onset within this group. These results are further corroborated by the conducted MCA and a recent smaller scale study [38]. Conversely, professionals selected more “general” onset symptoms regardless of the specific CDG, highlighting the heterogeneity of CDG. Interestingly, neither families nor professionals identified liver issues and blood abnormalities, such as coagulation problems, as primary onset symptoms (> 50%). For liver involvement, patient reports ranged from severe liver failure to milder instances of elevated serum transaminases. This variation in liver-related issues across different disorders suggests that liver involvement might not be a consistent feature during the onset and suspicion of CDG [53]. As for hematological involvement in CDG patients, despite its frequent occurrence in PMM2-CDG [54], it’s often overlooked in many case reports [55]. These problems may only be recognized after a thrombotic event or during routine lab tests before surgery [56]. As a result, both these systems might not be recognized as standout, unique, or primary symptoms at the onset of the condition.

In terms of symptom onset timing, a considerable portion of the respondents stated that their quest to get a definitive diagnosis was approximately a year. This still signifies a shift towards shorter diagnostic timelines in recent years, as displayed from older to new reports [29, 38, 51, 57–59]. The increased use of Whole Genome and Whole Exome Sequencing (WGS and WES) may contribute to this trend and could potentially make the term “diagnostic odyssey” obsolete in the future [60–62]. The temporal reduction to get a diagnosis reflects a decline in financial expenditure and the emotional strain associated with the diagnostic odyssey. It enables better care and disease management, ultimately improving the quality of life for individuals with CDG and their families.

In the journey of diagnosing CDG, non-PMM2-CDG patients face more misdiagnoses than PMM2-CDG counterparts, likely due to the latter’s higher prevalence and research depth. This highlights the need for improved knowledge on non-PMM2-CDG. Factors hindering this include geographic dispersion, absence of disease models, and growing CDG varieties.

When analyzing this group of rare diseases from a geographical perspective, there is a potential delay in diagnosis in Latin America compared to Europe and North America. These differences may be due to variations in rare disease policies. The US and the European Union have implemented successful initiatives, such as the US Rare Diseases Act of 2002 [63], which established the Rare Diseases Clinical Research Network, and the EU’s efforts starting with the Commission Communication in 2008, the Council Recommendation in 2009, and the Directive on Cross-border Healthcare in 2011, which improved care for rare diseases [64]. In 2010, Colombia was the first Latin American country to pass a national legislation for rare diseases, recognizing them as a public health issue. Other Latin countries followed their example like Brazil with its “National Policy for Rare Diseases” in 2014 [65]. However, as in some developed countries, effective implementation of these policies remains a challenge. Furthermore, there is a lack of funding and support for treating people living with rare diseases in these countries. The absence of established centers dedicated to studying rare diseases, such as PMM2-CDG and other less frequent CDG, makes the quest for a diagnosis even more difficult. This lack of infrastructure often forces patients to seek genetic testing abroad to obtain a definitive diagnosis, adding to the burden on families and delaying treatment or management therapies. Fortunately, local associations, such as Casa Hunter in Brazil and Federación Colombiana de Enfermedades Raras in Colombia, have been crucial in supporting rare disease treatment and advocating for better policies [66].

Despite the establishment of CDG international initiatives such as the EUROGLYCAN-omics, MetabERN and FCDGC, as well as the implementation of rare disease policies, misdiagnoses and undiagnosed persist [67–70]. On one hand, even among specialists studying CDG, new variants of these disorders are continually being discovered. Therefore, unresolved cases could possibly be attributed to genes and/or genotypes (pathogenic variants) not yet recognized as a CDG. In the near future, a turning point to this matter could be the widespread implementation of AI-based diagnostic tools which have the potential to assist in identifying patients with rare diseases like CDG [71, 72]. On the other hand, limited knowledge of rare diseases among healthcare professionals continues to be one of the most significant hurdles. Professionals not specialized in CDG may not provide sufficient information to families at the time of diagnosis, a concern voiced by numerous families in our study and supported by the findings of De Freitas et al. [73]. Potentially, due to their lack of knowledge of the disease and its complexity. To address this, it is essential to both educate healthcare professionals and empower families.

Rare disease education is insufficient in many countries [74–77], leading entities like the European Union to prioritize the development of rare disease expert networks, as outlined in their 2008 EUROPLAN, which remains relevant today through the European Reference Networks (ERNs) [78, 79]. Healthcare providers, are often the primary source of information at diagnosis as seen also by our results. However, their lack of information force families to depend on one another, assuming the role of expert, which explains the observed temporal shift concerning the source of information and their valuable information role in the community. We align with Kopeć et al. [80]. proposition for a comprehensive, potentially mandatory curriculum integrated into the routine medical training. Such a change in education is expected to improve their ability to understand and manage rare diseases. For in-service physicians, we recommend the adoption of a continuing education approach, facilitated by CDG experts and patient organizations, involving seminars and other educational activities. These includes the integration of global multidisciplinary hybrid forums on rare diseases. This type of event could be aligned with, for example, the U.S. Continuing Medical Education (CME) system. Such a model not only facilitates valuable insights into rare diseases for healthcare professionals worldwide but also aligns with and enhances continuing education requirements across various countries [81]. Additionally, educational materials should also be developed and shared with medical units that have potentially a higher prevalence of specialists in contact with CDG patients, such as geneticists, neurologists, and pediatricians. Examples of such materials have already been created by POs like CDG Care, APCDG and the World CDG Organization, among others [82, 83]. This strategy aims to provide extensive guidance, accelerate definitive diagnosis within the healthcare journey, and swiftly link them with families who have experienced similar situations.

Families can be empowered by different approaches, namely the establishment of POs and social media networks. These platforms provide support, facilitate connections between families, and disseminate the latest information on CDG as it was pointed out from their potentials by both professionals and families. The establishment of CDG families’ private Facebook groups is one such prime example that aims to achieve these goals. Furthermore, renowned CDG POs (e.g., CDG Care, APCDG, CDG Canada, CDG UK, among others) have made significant contributions to the CDG community by:

1. Co-organizing national and international conferences (e.g. World Conference on CDG, Rare Disease Day Symposium and CDG/NGLY1 Family Conference); as well as Q&A sessions with leading experts;
2. Developing websites (e.g., WorldCDG.org [83], CDG Care [84], CDG Hub [11]) which provide information on specific CDG in printed materials (e.g., pamphlet, infographics, newsletters) or web sections formats [85];
3. Conducting community-driven research by disseminating and developing patient registries, like CDG Connect as well as the work developed by the CDG & Allies research network, and fundraising research in CDG [86–88];
4. Awareness campaigns: Rare Disease Day (28th February), World CDG Awareness Day (16th May);
5. Providing financial support for families to cover costs related to attending CDG-focused conferences, traveling to clinical trial sites, and scheduling appointments with leading CDG experts [84].

Lastly, this questionnaire exemplifies community-driven research and family empowerment by recognizing the vital role that families occupy as valuable sources of information in community-centered investigations. As also demonstrated in previous studies [29, 32, 89, 90].

### Study strengths, biases and limitations

This study tries to provide a comprehensive investigation into the experiences and needs of individuals with CDG and their families, from symptom onset to definitive diagnosis and the ensuing informational requirements. While this research constitutes a pioneering effort in the field, to the best of our knowledge, it is not without its limitations.

As previously described by Francisco et al. [30], the cohort examined in the present study is characterized by a disproportionately low number of self-identified CDG expert professionals relative to the number of families. This imbalance might be due to a potential selection bias, since our distribution strategy targeted a network of contacts already involved in the CDG field, limiting our reach to the larger professional community. As a result, we did not include questions about professional awareness levels. Furthermore, the high academic qualifications of the participants may introduce bias, resulting in an analysis that only reflects a specific subset of the CDG population. Another factor to consider is our distribution strategy. We primarily used POs to spread the survey, so we mainly reached out to families who follow these groups. Additionally, our reliance on social media networks for disseminating the survey potentially skews our sample towards those with more digital literacy and more active on these platforms, possibly overrepresenting their perspectives and experience. Consequently, the generalizability of the findings may be limited.

Another confounding factor is the inherent variability in healthcare systems among countries. This heterogeneity may contribute to disparities in patient journeys, necessitating caution when interpreting the results. Additionally, the overrepresentation of PMM2-CDG families in the sample may skew the overall characterization of the patient journey, potentially obscuring the gaps in knowledge and experience for other CDG. Moreover, because CDG children with a more severe phenotype have a high mortality rate, their caregivers may not pass on their information into this questionnaire due to the lack of clarity to the inclusiveness of deceased patients in this study. These children are probably presenting a myriad of signs and symptoms at an earlier onset. Conversely, families with very mild phenotypes may be underrepresented in our study, as these families may not be as actively involved with CDG-related initiatives. Also, due to the lack of patients answers to the questionnaire, most likely due to the nature of these group of diseases, we are not able to create a single group composed solely of patients. Therefore, we are not able directly assess some of their personal needs apart from the ones pointed out by their parents/caregivers. These biases could potentially skew our overall results and our understanding of the spectrum of CDG severity.

On the other hand, ‘Additional Files’ were developed detailing the differences in the journey of each CDG. It is worth noting that the lack of centralized information on misdiagnosis for each CDG complicates comparisons with the literature. Further research could address this issue by conducting a more systematic investigation of misdiagnoses. Since there was a major focus on the diagnostic journey, there is a need to characterize the rest of the CDG journey that patients still have to live namely the management of the disease, which encompass clinical therapy, dietary options, hospitalizations, medications, of many. Also, this study does not encompass the journey of some CDG, such as GNE-CDG, as for instance this particular CDG typically manifests later in life than other CDG [91].

## Conclusion

In conclusion, the CDG Journey Mapping questionnaire offers a comprehensive perspective on the complex trajectory of individuals with CDG and their families, from symptom onset through diagnosis and beyond. Our investigation accentuates the obstacles confronted by families during the diagnostic odyssey, stressing the necessity of educating healthcare professionals and empowering families. The heterogeneity of CDG and the need for increased research extending beyond PMM2-CDG pose substantial challenges to augmenting comprehension and reducing misdiagnoses. Nonetheless, initiatives such as the Undiagnosed Diseases Network International, the worldwide CDG organizations, FCDGC, MetabERN, EUROGLYCAN-omics furnish hope and support for the community.

While this study provides valuable preliminary insights into the unique challenges faced by CDG families, additional research is indispensable to surmount the limitations and broaden the extant understanding. This would enable healthcare professionals and policymakers to devise more targeted interventions and support systems, ultimately ameliorating the lives of those impacted by CDG and their families. In essence, this investigation emphasizes yet again the crucial role of community-centered research and the invaluable insights that families can contribute to the pursuit of improving the diagnosis of and care for CDG.

## Electronic supplementary material

Below is the link to the electronic supplementary material.

Supplementary Material 1

Supplementary Material 2

Supplementary Material 3

Supplementary Material 4

Supplementary Material 5

## Data Availability

All data generated or analysed during this study is included in this published article. (and its additional files).
