# Supplementary material for: Mapping the diagnostic odyssey of congenital disorders of glycosylation (CDG): insights from the community
Source: Orphanet J Rare Dis. 2024 Nov 1;19:407. doi: 10.1186/s13023-024-03389-2 (PMC11529564; doi:10.1186/s13023-024-03389-2)
Supplement: Supplementary file 2 — Supplementary Material 2 [file 13023_2024_3389_MOESM2_ESM.pdf]

CDG JOURNEY MAP SURVEY FROM PROFESSIONALS VIEWS

(PROFESSIONALS VERSION)

INTRODUCTION

From the perspective of “community-centered” healthcare, mapping professionals' perceptions of how people living with a certain condition navigate throughout their journey is critical to improve the healthcare pathway.

OBJECTIVE OF THE SURVEY

The present study corresponds to part 2 of an international study aiming to capture the full picture on how people living with a Congenital Disorder of Glycosylation (CDG) navigate throughout their journey from different perspectives: the ones living with CDG, their caregivers and the healthcare professionals involved. This survey has the specific goals:

- To describe the experiences during the quest for CDG diagnosis, and identify CDG-related information needs throughout the CDG journey (since diagnosis),
- To map the level of awareness about the development and dissemination of Clinical Guidelines (CGs) for CDG,
- To collect experiences on participation in CDG clinical research
- To identify current support measures and gaps given by Worldwide CDG patient groups,
- To assess digital solutions tailored for the CDG community.

Why this study?

Our research team has identified a major gap in current CDG knowledge. The true CDG journey is still unknown. We have all heard about family’s experiences, firsthand narratives, and medical reports that emerge related to the quest for diagnosis and the struggle for appropriate care. But to our knowledge no study has systematically collected the CDG journey. Mapping CDG experience along the entirety of their disease pathway, from initial signs and symptoms through diagnostics to first definitive treatment, enables a greater understanding of the efficiency of care delivery and identifies opportunities for improvement or, in the case of CDG management, any points that require further clarity or additional interventions.

DISSEMINATION STRATEGY OF THE RESULTS

- Be published in different formats to allow their use for advocacy and policy making at international levels (we plan to publish an article, fact sheets and infographics).
- Better define the need for resources, and allow for better tailored initiatives for people who live with CDG, healthcare professionals or stakeholders’ individual needs, helping the person who lives with a certain disease experience and leading to improved individuals outcomes.

DISCLAIMER

If you need any help or additional information about the questionnaire or clarifications on the content, please do not hesitate to contact us at https://worldcdg.org/contact, where the authors of this study Paula Videira or Vanessa dos Reis will be available to schedule a SKYPE, WhatsApp CALL, or Zoom meeting.

Can the survey be saved and finished later?
YES. Participants can fill in part of it and then return to it to finish it, using the same device (e.g. mobile phone, laptop) and internet browser. To return to the previous question, please hit the PREV BUTTON, instead of the back button from the internet browser.

Clicking on the "agree" button below indicates that:

- You voluntarily agree to participate;
- You are a healthcare professional or from a related background;
- The estimated time to complete the whole survey is **37 minutes**;

You understand that the responses to the survey are anonymous and that no information will be collected or processed that allows your identification.

THEME - PARTICIPANT RELATIONSHIP WITH CDG

1. Please indicate your choice: ○ Agree (Q2) ○ Disagree (End of the Survey)

2. Questionnaire completed by (please choose in which capacity you want to answer this questionnaire. If more than one choice qualifies, please check the most appropriate).

- ○ Pharmaceutical or biotech representative
- ○ Researcher/Clinicians
- ○ A CDG healthcare professional or other professional support provider

THEME - THE JOURNEY FROM FIRST SIGNS TO A FINAL DIAGNOSIS

3. At which age do families on average report that CDG symptoms were first manifested in their CDG child/adult? **(Please check the most appropriate option)**

- ○ Antenatally/Prenatally
- ○ < 3 months
- ○ 3 - 6 months
- ○ 7 - 9 months
- ○ 10 - 12 months
- ○ 1- 3 years old
- ○ 4 - 9 years old
- ○ 10 - 17 years old
- ○ 18 - 21 years old
- ○ > 21 years old
- ○ I'm a basic researcher, I don't follow patients
- ○ I don' know

4. What were the main CDG presenting signs and symptoms (refer to the manifestations that **first led** you or your clinician to **suspect something** was wrong)? **(Please select all that apply)**

- ○ Low muscle tone or floppiness
- ○ Poor growth
- ○ Failure to thrive
- ○ Developmental disabilities
- ○ Liver disease and/or with elevated liver enzymes
- ○ Abnormal bleeding or blood clotting
- ○ Bone manifestations
- ○ Abnormal brain imaging
- ○ Abnormal lab tests
- ○ Poor night vision and loss of peripheral vision
- ○ Recurrent infections
- ○ Feeding problems
- ○ Strabismus
- ○ Seizures
- ○ Stroke-like episodes
- ○ Heart problems
- ○ Ataxia
- ○ Slurred speech (dysarthria)
- ○ I'm a basic researcher, I don't follow patients
- ○ Other

**5. In your experience, how many doctors (approximately) a person that lives with CDG and their family members... (Please select the most appropriate option)**	1 - 2	3 - 5	6 - 10	11 - 20	> 20	I don't know	Basic Researcher
consult when they SUSPECTED of their FIRST SIGNS AND SYMPTOMS of CDG?	○	○	○	○	○	○	○
consult between the first manifestations and their FINAL CDG diagnosis?	○	○	○	○	○	○	○

(RATING SCALE)

6. How much time (approximately) usually a person who lives with CDG takes to get their FINAL CDG diagnosis? **(Please select the most appropriate option)**

- < 3 months
- 3 - 6 months
- 7 - 12 months
- 1- 3 years old
- 4 - 5 years old
- 6 - 9 years old
- 10 - 20 years old
- > 20 years old (please specify)
- I'm a basic researcher, I don't follow patients
- I don't know

7. In your experience, is it common for a person who lives with CDG to be misdiagnosed, before their FINAL diagnosis is made?

- Yes (Q11)
- No (Q12)
- I don't know (Q12)
- I'm a basic researcher, I don't follow patients

8. Which diagnoses are usually given before the final diagnosis? **Note: If you do not know, please write "I don't know".**

9. In your experience, concerning the medical specialties involved, please tell us the **FIRST to raise the possibility of a CDG diagnosis**

- Geneticist
- Neurologist
- Paediatrician
- Metabolic specialist
- Obstetrician
- General practitioner
- Cardiologist
- Endocrinologist
- Gastroenterologist
- Ophthalmologist
- I don't know
- I'm a basic researcher, I don't follow patients
- Other (please specify)

10. In your experience, concerning the medical specialties involved, please tell us the **specialist who gave the FINAL diagnosis**

- Geneticist
- Neurologist
- Paediatrician
- Metabolic specialist
- Obstetrician
- General practitioner
- Cardiologist
- Endocrinologist
- Gastroenterologist
- Ophthalmologist
- I don't know
- I'm a basic researcher, I don't follow patients
- Other (please specify)

THEME - ACCESS TO INFORMATION ABOUT CDG

11. Which format of information about CDG do you usually provide to families? Please select all that apply.

- Printed material (e.g. brochures, leaflet)
- Referred to website
- Social media
- Journal Article
- Verbal
- I'm a basic researcher, I don't follow patients
- Other

THEME - SUPPORT FOR FAMILIES THROUGHOUT PATIENT GROUPS

12. Are you aware of any organisation or support group specific to CDG?

- Yes
- No
- I don't know

13. In your opinion, how important is the role of patient organisations in providing information about CDG to affected people and families?

Not very important | Not important | Important | Very Important | Essential

THEME - CDG-FOCUSED DIGITAL SOLUTIONS THAT MEET THE NEEDS OF THE PEOPLE WHO LIVE WITH CDG

14. Are you on Social media platforms (Facebook, Twitter, and others etc)?

- Yes (Q37)
- No (Q39)

15. Are you already familiar with any active social media groups focused on CDG that you consider as good examples?

- Yes
- No
- I don't know

16. What do you think social media can/could do for the CDG community? **(Please select all that apply)**

- Raise CDG awareness
- Connect different stakeholders worldwide
- Learn about clinical trials
- Optimise Clinical trials (lowering its costs, time, etc)
- Help CDG families at the time of diagnosis
- Creation of CDG worldwide organizations
- Broader CDG information
- Promote resources of interest for CDG community
- Get advice of fellow people who live with the same condition
- Learn about the latest research news
- Fundraise for CDG
- To secure a rare disease patient perspective into broader online conversations
- Other (please specify)

THEME - SOCIODEMOGRAPHICS INFORMATION

17. What is your age?

- 18 - 24 years old
- 25 - 34 years old
- 35 - 44 years old
- 45 - 54 years old
- 55 - 65 years old
- Above

18. Are you :

- Male
- Female
- Other (Please specify)

19. In which country do you live?

- Afghanistan
- ......
- Zimbabwe

20. What is your highest qualification?

- Less than high school diploma
- High school diploma or equivalent degree
- Bachelor’s degree
- Master's degree
- PhD
- Other (Please specify)

Thank you for participating in this survey !

Click on **DONE** - at the end of this page - to **FINISH**.

We would be very grateful if you could share a link to https://worldcdg.org/research-cdg-journey-mapping/survey-2-cdg-experiences-over-time-families-and-professionals-views on your Facebook and Twitter pages to allow your followers to join and take part in the surveys.

In accordance with the Data Protection laws, you can access, modify, or suppress your information at any time. If you want to exercise this right and obtain information about your data, please contact https://worldcdg.org/contact

ACKNOWLEDGMENTS

CDG & Allies – Professionals and Patient Associations International Network (CDG & Allies – PPAIN) deeply acknowledge the participation of the stakeholders that acted as advisors in this survey.

Thank you for taking time to participate in our questionnaire.

We truly value the information you have provided.

The information gained from this survey will be valuable in developing better health and supports services for children with CDG, benefiting both them and their families.

Copyright © APCDG & CDG & Allies – PPAIN, Portugal 2021. All rights reserved. No part of this questionnaire may be reproduced, distributed, or transmitted in any form or by any means, including photocopying, recording, or other electronic or mechanical methods, without the prior written permission of the publisher, except in the case of brief quotations embodied in critical reviews or related publications and certain other noncommercial uses permitted by copyright law.

SOURCES

1. Zurynski, Y., Deverell, M., Dalkeith, T. et al. (2017). Australian children living with rare diseases: experiences of diagnosis and perceived consequences of diagnostic delays. Orphanet J Rare Dis 12, 68. https://doi.org/10.1186/s13023-017-0622-4
2. Crowe, A. L., McKnight, A. J., & McAneney, H. (2019). Communication Needs for Individuals With Rare Diseases Within and Around the Healthcare System of Northern Ireland. Frontiers in public health, 7, 236. https://doi.org/10.3389/fpubh.2019.00236
3. Franco JVA, Arancibia M, Meza N, Madrid E, Kopitowski K. (2020). Clinical practice guidelines: Concepts, limitations and challenges. Medwave. 20(3):e7887. Spanish, English. doi: 10.5867/medwave.2020.03.7887. PMID: 32428925.
4. https://www.who.int/health-topics/clinical-trials/#tab=tab_1 Accessed April 2021
5. Davies, W. (2016) Insights into rare diseases from social media surveys. Orphanet J Rare Dis 11, 151. https://doi.org/10.1186/s13023-016-0532-x
