# Supplementary material for: Mapping the diagnostic odyssey of congenital disorders of glycosylation (CDG): insights from the community
Source: Orphanet J Rare Dis. 2024 Nov 1;19:407. doi: 10.1186/s13023-024-03389-2 (PMC11529564; doi:10.1186/s13023-024-03389-2)
Supplement: Supplementary file 5 — Supplementary Material 5 [file 13023_2024_3389_MOESM5_ESM.pptx]

## Slide 1
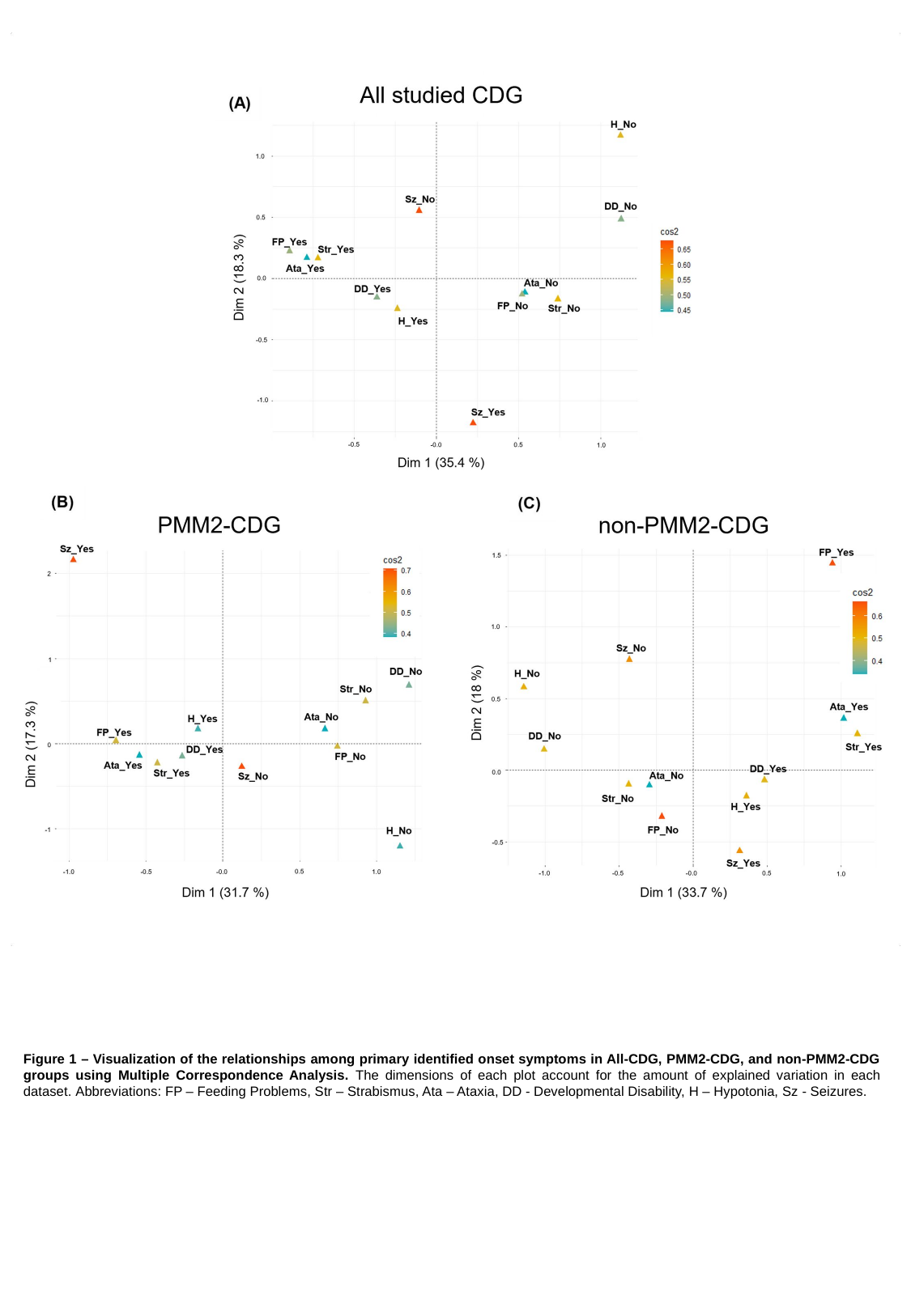

Figure 1 – Visualization of the relationships among primary identified onset symptoms in All-CDG, PMM2-CDG, and non-PMM2-CDG groups using Multiple Correspondence Analysis. The dimensions of each plot account for the amount of explained variation in each dataset. Abbreviations: FP – Feeding Problems, Str – Strabismus, Ata – Ataxia, DD - Developmental Disability, H – Hypotonia, Sz - Seizures.

## Slide 2
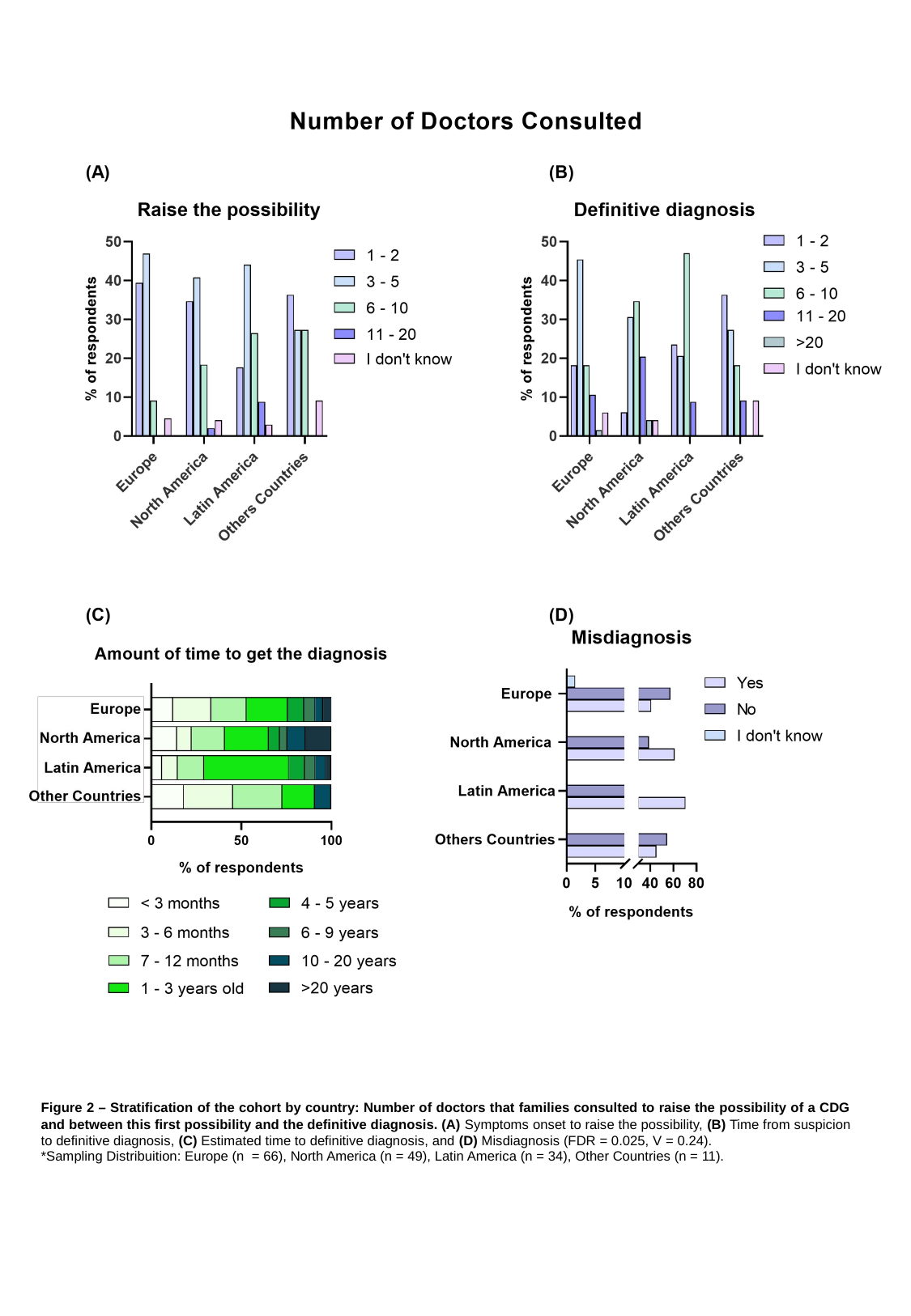

Figure 2 – Stratification of the cohort by country: Number of doctors that families consulted to raise the possibility of a CDG and between this first possibility and the definitive diagnosis. (A) Symptoms onset to raise the possibility, (B) Time from suspicion to definitive diagnosis, (C) Estimated time to definitive diagnosis, and (D) Misdiagnosis (FDR = 0.025, V = 0.24).
*Sampling Distribuition: Europe (n = 66), North America (n = 49), Latin America (n = 34), Other Countries (n = 11).

## Slide 3
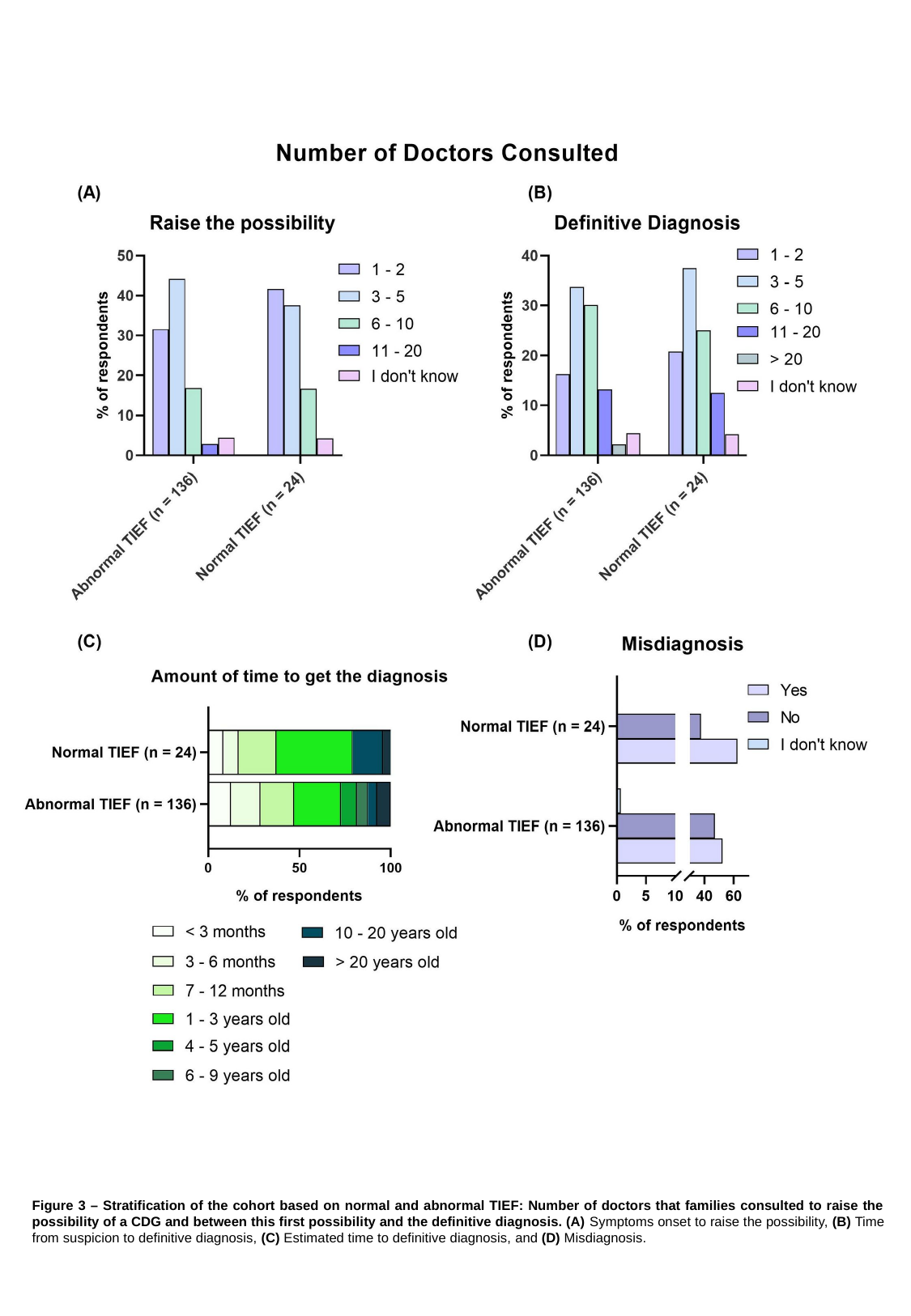

Figure 3 – Stratification of the cohort based on normal and abnormal TIEF: Number of doctors that families consulted to raise the possibility of a CDG and between this first possibility and the definitive diagnosis. (A) Symptoms onset to raise the possibility, (B) Time from suspicion to definitive diagnosis, (C) Estimated time to definitive diagnosis, and (D) Misdiagnosis.

## Slide 4
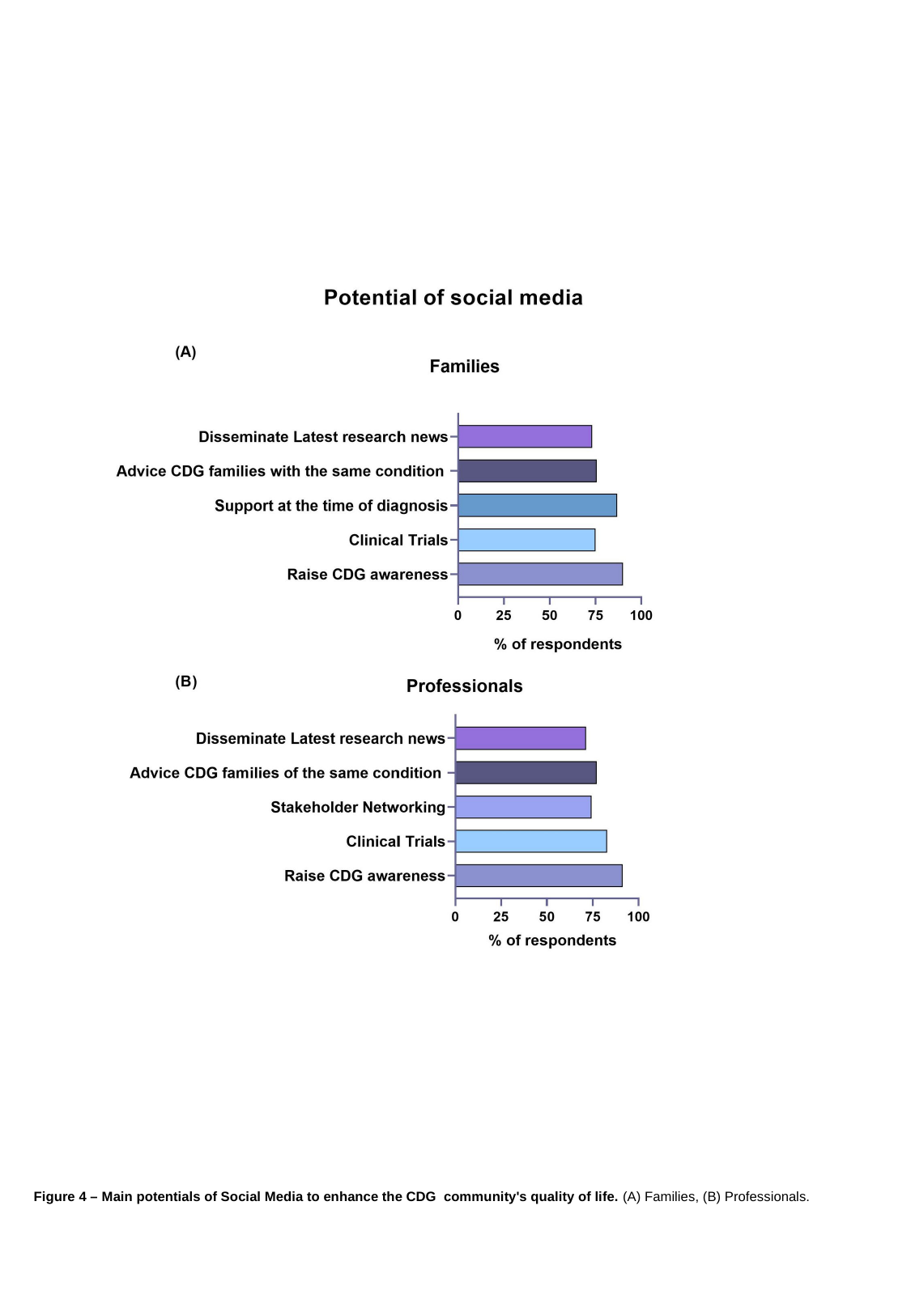

Figure 4 – Main potentials of Social Media to enhance the CDG  community's quality of life. (A) Families, (B) Professionals.
